# On the Treatment of Aneurism by Compression

**Published:** 1846-08

**Authors:** 

**Affiliations:** Consulting Surgeon to the City of Dublin Hospital, &c. &c.


### SELECTIONS

FROM

### AMERICAN AND FOREIGN JOURNALS.

---

*On the Treatment of Aneurism by Compression.* By Dr. Porter, Consulting Surgeon to the City of Dublin Hospital, &c. &c.—We do not, says he, extol the practice as being simple and safe, and free from pain, and exempt from trouble; we only compare it with another, and advocate it on the grounds of being more free from danger, more conservative of human life. True, the disease may occupy situations where this particular mode of treatment cannot be adopted, to which there may be no means of applying pressure, or where there may not be sufficient space for its adaptation, and, therefore, we cannot forego our operations on the carotid, the subclavian, and the iliac arteries; but for the other forms of aneurism, and especially that formidable one, the popliteal, still more formidable because of its extreme frequency and the fatality which attended its former treatment, we ought to consider ourselves happy in possessing a resource which promises to supersede the necessity of an operation altogether. I look forward with hope, and indeed with confidence, to the day when it shall cease to hold a place in operative surgery, when it shall have become (at least in Ireland) a matter of professional history—the practice of by-gone days—and when a surgeon shall undertake the management of a case of popliteal aneurism with as much calmness and reliance on the resources of his art, as he lately approached it with apprehension.

Wm. Devereux, a tailor, æt. 29, was admitted into the Meath Hospital, December 3d, 1844, when he stated that he had generally enjoyed excellent health, until about two months previous to his application for relief, when he observed an unusual pulsation or throbbing in the right ham, which, however, gave him no uneasiness, except that he imagined that limb to be occasionally stiff in walking. He thus continued for six weeks, at the end of which a tumor appeared on the spot where he first observed the throbbing, which gradually increased until it attained the size of a small orange, though still unattended with pain; but the size of the swelling, and the stiffness produced by it, interfering with the power of extending the limb, he applied sixteen leeches, which he imagined had some effect in reducing its dimensions. During the last two days it increased so rapidly as to create alarm, and induce him to apply for assistance.

On examination, a tumor was observed pulsating strongly on the right popliteal space, and occupying its entire extent; firm and incompressible externally, but softer internally, where pressure had the effect of diminishing its volume considerably. The expansion during its diastole was equally perceptible in every direction, and a low *bruit de soufflet* was heard over every part of it. Pressure on the artery at the groin reduced its size by nearly one-third. The man's general state of health appeared to be very good, and he made no complaint except of pain occasionally shooting down to the external ancle, particularly at night.

Dec. 6. At half past ten o'clock this morning I applied pressure on the trunk of the femeral artery by means of two clamps; one applied about two inches below Pouparts' ligament, the other at a spot nearly corresponding to the place where the vessel entered the tendon of the triceps muscle, with the intention that one might be screwed down when it became necessary that the other should be relaxed, and thus a constant and uninterrupted compression be maintained. The superior clamp was then screwed down until the impulse of the circulation was communicated to the instrument, which vibrated with each stroke of the vessel. Under this pressure the pulsation of the tumour was scarcely, if at all, diminished, and he bore it without apparent inconvenience for a quarter of an hour, when it was relaxed—the inferior clamp having been screwed so as to produce a corresponding effect: but it was found that he could not endure this latter either so well or for such a length of time as the former, the reason of which appeared to be that a comparatively much greater force was necessary to produce a given effect on the circulation when used where the vessel was deeply covered, and consequently the pressure on the soft parts there was stronger and less endurable. In the evening the limb was slightly swollen and discolored, and he complained of pain in the calf of the leg, which latter symptom was relieved by wrapping the part in flannel.

Dec. 7. Passed a sleepless night; the limb more swollen but less painful.

Dec. 8. Had some sleep from the effects of an opiate. The pain was now trifling, but the tumefaction increased, and there was some œdema. On this day he was able to endure the pressure of the superior clamp for half an hour, and of the inferior twenty minutes; neither instrument was ever screwed so tightly as completely to obstruct the circulation, or stop the pulsation in the tumor.

Dec. 10. On this morning I found both clamps loose and unscrewed; this had been done by the patient himself, who was feverish, irritable, and discontented, complaining of head-ache, and having a hot skin and accelerated pulse. He was ordered some aperient medicine, which afforded great relief.

Dec. 12. I found it nearly impossible to affect the circulation of the limb by the inferior clamp: it could not be brought to bear directly on the vessel, and consequently the degree of pressure used was too forcible without being very effective. Mr. Millikin, the ingenious instrument maker, of Parliament street, contrived a clamp in which this objection was obviated; it answered the purpose admirably.

Dec. 13. The œdema of the limb had nearly disappeared, and there was no pain; the *bruit de soufflet* scarcely perceptible in the tumor, and the force of pulsation diminished.

Dec. 15. The case seemed to be progressing most favorably, when, unfortunately, the new instrument broke, and pressure of any kind was discontinued for several hours. The symptoms all returned as in the commencement.

Dec. 16. A new instrument having been constructed, the pressure was resumed, the symptoms being as strongly marked as at any previous period, but the general condition of the patient much more favorable; he could now endure the operation of the superior clamp uninterruptedly for an hour and a half, that of the inferior one for an hour, and neither occasioned œdema, discoloration, or pain.

Dec. 20. The *bruit de soufflet* was no longer audible, and the tumor seemed to be firmer and smaller. The patient appeared perfectly to understand the object of the treatment, which had been frequently explained to the pupils at his bed side, and managed the screw of the instrument himself with considerable judgment: the necessary attendance on him was, of course, diminished, and the case afterwards gave but little trouble.

Dec. 30. On the evening of this day he suddenly exclaimed that the pulsation in the ham had ceased, and that he was cured. On examination such was found to be the case.

Jan. 6. The tumor was hard, diminished in size, and totally devoid of pulsation. He was up on this day, weak and somewhat emaciated from confinement, but in other respects perfectly well. He has since been frequently seen by me and several of the pupils of the hospital; and up to the last day on which he came under observation (July 19, 1845) he remained without the slightest apparent tendency to a recurrence of the disease. The limb that has been engaged exhibited no difference from the other, except that, on minute examination, a small, firm, kernel-like tumor could be felt deep in the popliteal space.—*Dublin Quarterly Jour. of Med. Science.*

